# Preface

**DOI:** 10.1155/DTE.5.vii

**Published:** 1999

**Authors:** Harubumi Kato


*Diagnostic and Therapeutic Endoscopy*, Vol. 5, p. vii
Reprints available directly from the publisher
Photocopying permitted by license only

© 1999 OPA (Overseas Publishers Association) N.V.
Published by license under
the Harwood Academic Publishers imprint,
part of The Gordon and Breach Publishing Group.
Printed in Singapore.

### Preface

Technologies in endoscopic diagnosis and therapy have advanced significantly due to recent remarkable progress of medical engineering. In the 1970's the diagnostic accuracy was improved markedly by the development of fiberoptic endoscopy which had just appeared in the late 1960's. In the 1980's endoscopic treatment with several kind of lasers in combination with endoscopy began. Since endoscopic, especially fiberoptic endoscopic, diagnosis and treatment do not require general anesthesia, these modalities can reduce the burden on the patient. Minimally invasive endoscopic diagnostic and therapeutic modalities will progress further in the future.

This special issue is devoted to recent developments in endoscopic diagnostic procedures.

The fluorescence endoscopy is one means to localize precancerous or cancer lesions. There are three approaches: photodynamic diagnosis by observation of fluorescence produced by a photodynamic reaction due to excitation of a tumor-specific photosensitizer by light, diagnosis based on the recognition of a defect in the autofluorescence image from lesions, and diagnosis by a combination of photodynamic and autofluorescence diagnosis.

Cancer mortality rates are increasing throughout the world. In particular the lung cancer, colon cancer and breast cancer mortality rates are increasing significantly because of many factors, including smoking, air pollution, the increasing average age of society and delay in detection. Lung cancer has one of the worst prognoses among malignant diseases. In order to control and reduce the mortality rate, early detection and localization are necessary.

However particularly in lung cancer, although sputum cytology is an effective method to detect central type early stage squamous cell carcinoma, there are still some problems.

One of these is the difficulty of the localization of extremely early stage lung cancer even with positive sputum cytology.

For such early lesions other technique such as spectroscopic imaging are required. These new developments have made it possible to localize otherwise invisible precancerous and early cancerous lesions. These technique hold future promise for more precise diagnosis of endoscopically curative early stage malignant tumors.

This issue provides a glimpse of the development of these new techniques and equipment with particular focus on bronchial and gastrointestinal lesions.

Harubumi Kato, M.D., Ph.D.
Editor-in-Chief

